# Inactivation of *L. monocytogenes* and *S. typhimurium* Biofilms by Means of an Air-Based Cold Atmospheric Plasma (CAP) System

**DOI:** 10.3390/foods9020157

**Published:** 2020-02-06

**Authors:** Marlies Govaert, Cindy Smet, Annika Graeffe, James L. Walsh, Jan F. M. Van Impe

**Affiliations:** 1 CPMF2—Flemish Cluster Predictive Microbiology in Foods—www.cpmf2.be, 9000 Ghent, Belgium; marlies.govaert@kuleuven.be (M.G.); cindy.smet@kuleuven.be (C.S.); 2 OPTEC—Optimization in Engineering Center-of-Excellence, KU Leuven, 9000 Ghent, Belgium; 3 BioTeC—Chemical and Biochemical Process Technology and Control, Department of Chemical Engineering, KU Leuven, 9000 Ghent, Belgium; annikag@freenet.de; 4 Department of Electrical Engineering and Electronics, University of Liverpool, Liverpool L69 3BX, UK; J.L.Walsh@liverpool.ac.uk

**Keywords:** Cold Atmospheric Plasma (CAP), inactivation, biofilms, *L. monocytogenes*, *S. typhimurium*, feed gas, electrode configuration

## Abstract

Previous (biofilm) inactivation studies using Cold Atmospheric Plasma (CAP) focused on helium (with or without the addition of oxygen) as feeding gas since this proved to result in a stable and uniform plasma. In industry, the use of helium gas is expensive and unsafe for employees. Ambient air is a possible substitute, provided that similar inactivation efficacies can be obtained. In this research, 1 and 7 day-old (single/dual-species) model biofilms containing *L. monocytogenes* and/or *S. typhimurium* cells were treated with an air-based Surface Barrier Discharge (SBD) plasma set-up for treatment times between 0 and 30 min. Afterwards, cell densities were quantified via viable plate counts, and predictive models were applied to determine the inactivation kinetics and the efficacy. Finally, the results were compared to previously obtained results using a helium-based SBD and DBD (Dielectric Barrier Discharge) system. This study has demonstrated that the efficacy of the air-based CAP treatment depended on the biofilm and population type, with log-reductions ranging between 1.5 and 2.5 log_10_(CFU/cm^2^). The inactivation efficacy was not significantly influenced by the working gas, although the values were generally higher for the air-based system. Finally, this study has demonstrated that the electrode configuration was more important than the working gas composition, with the DBD electrode being the most efficient.

## 1. Introduction

Biofilms are highly resistant consortia of cells which are embedded in a matrix of self-produced extracellular polymeric substances (EPS) and attached to a biotic or an abiotic surface [1,2,3,4]. They are omnipresent in nature and in industrial environments such as the food industry, causing economic and health-related problems such as contamination/spoilage of food products, an impeded heat transfer in heat exchangers, and corrosion of surfaces [3,5,6,7]. In order to avoid contamination of food products following contact with abiotic surfaces such as knives, conveyor belts, and packaging material, biofilms developed on these surfaces should be inactivated in an adequate way. Many studies proved, however, that currently used methods for the decontamination of abiotic surfaces (i.e., the use of (hot) water combined with antimicrobial agents and a mechanical action [8]) can be highly inefficient for biofilm inactivation [2,4,5,9]. Therefore, research towards novel (more effective) inactivation methods is increasing.

Cold Atmospheric Plasma (CAP) is one of these promising novel methods for biofilm inactivation as log-reductions up to 4 log_10_(CFU/cm^2^) have been obtained for inactivation of *L. monocytogenes* and *S. Typhimurium* (single/dual-species) biofilms (e.g., [10,11,12,13,14]). CAP is a specific type of plasma which can be created by the addition of energy to a gas at room temperature and at atmospheric pressure. This energy addition results in the creation of an energized gas which consists of a variety of reactive species such as ions (positive and negative), photons, free electrons, and activated neutral species (excited and radical) [15,16,17,18,19,20]. Within previously mentioned promising biofilm inactivation studies, helium was often used as a working gas since this proved to result in the creation of a stable, uniform, and reactive plasma [15,16]. However, as (i) the use of gas bottles is (relatively) unsafe for employees and (ii) the use of helium on an industrial scale is very expensive, more research is required to replace helium by another working gas. Ambient air would be a suitable substitute, as no gas bottles are needed and it is non-expensive. In addition, according to many studies (e.g., [18,21]), the presence of oxygen results in the generation of many reactive oxygen species (ROS), enabling the creation of a highly efficient plasma. This high efficacy was, however, not observed within the research of Govaert et al. [13], where the addition of a small amount of oxygen (i.e., 1%, *v/v*) to a helium feed gas resulted in a faster, but less efficient (based on total log-reductions) biofilm inactivation. Nevertheless, in previously mentioned research, it was hypothesized that the addition of a small amount of oxygen could have resulted in an altered ratio of ROS and RNS (reactive nitrogen species). As the study of Duan et al. [22] proved that RNS are able to penetrate further into biological tissue, the high nitrogen content of air could probably be of more importance to obtain a high biofilm inactivation efficacy. Therefore, more research is required to investigate the specific efficacy of an air-based plasma for biofilm inactivation. In this regard, it is important to study the inactivation kinetics in order to select the most optimal CAP treatment time.

In this research, three different (single/dual-species) model biofilms containing *L. monocytogenes* and/or *S. Typhimurium* cells were treated with an air-based plasma set-up. In addition, for each of these biofilm types, two different biofilm ages were tested (i.e., 1- and 7-day-old). The model biofilms were subjected to CAP treatment for different treatment times (0–30 min), viable plate counts were used to determine the (remaining) cell density of the (CAP treated) biofilms, and predictive models were applied to describe the inactivation kinetics and the efficacy of the CAP treatment. Finally, the results of this study were compared to previously obtained results using a helium-based plasma system for inactivation of similar model biofilms.

## 2. Materials and Methods

### 2.1. Experimental Design 

In this study, three different model biofilms (i.e., a single-species *L. monocytogenes* biofilm, a single-species *S. Typhimurium* biofilm, and a dual-species biofilm containing both previously mentioned species) were inactivated by means of an air-based CAP system. For each of the model biofilms, two different biofilm ages (i.e., 1- and 7-day-old) were used in order to comment on the effect of this biofilm characteristic on the CAP inactivation kinetics and efficacy. For the CAP treatment of the different model biofilms, a Surface Barrier Discharge (SBD) device was applied. Ambient air was used as operating gas, the input voltage was set at 24.88 V (which resulted in an average output voltage and an average dissipated power value of approximately 4 kV and 5.5 W, respectively), and the applied frequency was set at 12 kHz. Different treatment times (ranging between 0 and 30 min) were used and viable plate counts on non-selective/general and selective media were applied to determine the cell density of the biofilms before and after the CAP treatment. Predictive models were used to determine (i) the CAP inactivation kinetics and (ii) the efficacy of the treatment for each of the different model biofilms. Finally, the results obtained within this study were compared to previous research using a helium-operated CAP system for inactivation of similar single- and dual-species model biofilms [12,13,14]. Hence, the use of a small amount of previously obtained data can help to draw more general conclusions about the influence of certain plasma characteristics (i.e., the operating gas and the electrode configuration) on the biofilm inactivation efficacy of CAP.

### 2.2. Microorganism and Pre-Culture Conditions 

In this research, *L. monocytogenes* LMG23775 (isolated from sausages) and *S. Typhimurium* LMG14933 (isolated from bovine liver) were used. These strains were both acquired from the Belgian Co-ordinated Collections of Micro-organisms/Laboratory of Microbiology (BCCM/LMG) of Ghent University in Belgium. The authors refer to the research of Govaert et al. [23] for more information regarding the required conditions to obtain stationary phase pre-cultures with a cell density of approximately 10^9^ CFU/mL.

### 2.3. Biofilm Development Conditions 

The obtained stationary phase pre-cultures were used to develop 100-fold diluted inocula with a cell density of approximately 10^7^ CFU/mL. Dependent on the specific model biofilm, different dilution media were used. For the single-species *L. monocytogenes* and *S. Typhimurium* biofilms, Brain Heart Infusion broth (BHI, VWR International, Oud-Heverlee, Belgium) and 20-fold diluted Tryptic Soy Broth (TSB/20, Becton Dickinson, Holdrege, NE, USA) were used, respectively, since these media proved to be optimal for the development of strongly adherent and mature single-species model biofilms [23]. Based on the study of Govaert et al. [14], TSB/20 was also used as optimal dilution medium for the development of the dual-species model biofilms. Finally, to prepare the single-species inoculum, 100 µL of the *L. monocytogenes* or *S. Typhimurium* pre-culture was transferred to 10 mL of the appropriate dilution medium. For the dual-species biofilm, on the other hand, individual pre-cultures were mixed (at a volume ratio of 1:1) prior to transferring 100 µL of this mixture to 10 mL of the appropriate dilution medium.

To develop the biofilms, 1.2 mL of the appropriate inoculum was transferred to a small polystyrene Petri dish (50 mm diameter, 9 mm height, Simport, Saint-Mathieu-de-Beloeil, QC, Canada). After inoculation, the Petri dishes were closed and gently shaken to make sure the inoculum covered the entire surface. Dependent on the model biofilm and the biofilm age, Petri dishes were incubated for 1 or 7 day(s) at 25 or 30 °C. The former temperature proved to be optimal for development of (i) single-species *S. Typhimurium* biofilms and (ii) dual-species biofilms [14,23], while the latter temperature was optimal for *L. monocytogenes* single-species biofilm formation [23].

### 2.4. CAP Equipment and Biofilm Inactivation Procedure 

The air-based CAP system used for inactivation of the biofilms consisted of a high voltage power source capable of producing a sinusoidal waveform at a frequency of 12 kHz and an input voltage of 24.88 V, which resulted in an average output voltage of approximately 4 kV and an average dissipated power value of approximately 5.5 W. The output of the power source was connected to an SBD electrode configuration which consisted of metal strips adhered to the surface of an aluminum oxide dielectric sheet and which were embedded in epoxy to provide electrical shielding. The opposite side of the alumina sheet contained the counter electrode, which consisted of multiple metallic strips which were connected directly to ground. A sufficiently high electric field occurs at the electrode edges to cause ionisation of the applied working gas (i.e., ambient air), resulting in the formation of an air-based plasma which propagates along the dielectric surface.

As mentioned before, the results obtained within the presented research were compared to those previously observed using a helium-based set-up [12,13,14]. Within these studies, two electrode configurations were used, i.e., an SBD and a DBD (Dielectric Barrier Discharge) electrode. For both configurations, the frequency and the input voltage were set at 15 kHz and 21.88 V, respectively. For the SBD configuration, this resulted in an output voltage of 3.5 kV and a dissipated plasma power of 3.2 W, while for the DBD configuration, these parameters reached values of 6.4 kV and 7.0 W, respectively. For more specifications about the helium-based system, the authors refer to the study of Govaert et al. [13].

For the (air-based) CAP treatment within the presented study, rinsed and dried biofilm samples (see Section 2.5) were placed inside the electrode chamber underneath the electrodes (distance between electrodes and sample = ± 1.5 cm). After this, the electrode chamber was closed and the high-voltage power source was energized to generate the plasma. Samples were treated up to 30 min and immediately after the treatment removed from the reactor chamber to determine the remaining cell density (see Section 2.5). It should be mentioned, however, that the plasma system was operated empty for 20 min prior to the first treatment since preliminary research indicated that this resulted in a more stable plasma. In addition, in between samples, a waiting period was included in order to decrease the electrode temperature and to limit the temperature effect of the plasma treatment since the temperature of the Petri dish surface became approximately 35.5 °C following 30 min of CAP treatment. This waiting period had a similar duration as the treatment time applied for the former biofilm sample.

For the CAP treatment used within the studies of Govaert et al. [12,13,14], using a different type of set-up, this waiting period was not needed. However, prior to each treatment, the electrode chamber was flushed for 2 min (SBD) or 4 min (DBD) to generate a homogeneous (helium) environment.

### 2.5. Quantification of Biofilm Cell Density by Means of Viable Plate Counts 

Following 1 or 7 day(s) of incubation, biofilms were removed from the incubator and three times rinsed with 1.2 mL of Phosphate Buffered Saline (PBS) solution to remove the remaining planktonic cells. After the rinsing procedure, biofilms were allowed to dry in the laminar flow cabinet for approximately 30 min. Next, the biofilms were either immediately quantified (untreated biofilms) or first CAP treated (see Section 2.4). For the quantification, 2 mL of sterile PBS solution was added to the (un)treated sample and the biofilm was removed from the surface using a cell scraper (blade width 20 mm, Carl Roth GmbH + Co., Karlsruhe, Germany). The obtained cell suspension was transferred to an empty sterile micro-centrifuge tube, vortexed, and serial decimal dilutions were prepared (0.85 (*v/v*) % NaCl, Sigma-Aldrich, St. Louis, MO, USA). For each of the serial dilutions, three drops of 20 µL were plated on non-selective/general and selective media. Nevertheless, for samples containing few cells, drops of 100 µL were plated. Therefore, the final detection limit was 1.0 log_10_(CFU/cm^2^).

For the single-species biofilms, the cell density determined on the selective medium represents the healthy cell population, while both healthy and injured cells can grow on the non-selective/general medium. Based on the difference in colony forming units (CFU) between the non-selective/general and the selective medium, the percentage of sub-lethally injured cells was determined (see Section 2.6). For the dual-species biofilm, the cell density determined on the non-selective/general medium represents the total population, while the selective media were used to quantify the cell density of each individual population (i.e., *L. monocytogenes* and *S. Typhimurium*). For the *L. monocytogenes* single-species biofilm, Brain Heart Infusion agar (BHIA, BHI supplemented with 14 g/L biological agar, VWR International, Oud-Heverlee, Belgium) and PALCAM (VWR Chemicals, Belgium) were used as non-selective/general and selective medium, respectively. For the *S. Typhimurium* single-species biofilm, on the other hand, the applied non-selective/general and selective medium were Tryptic Soy Agar (TSA) and Xylose Lysine Deoxycholate agar (XLD, Merck and Co., Kenilworth, NJ, USA), respectively. Finally, for the dual-species biofilm, BHIA was used as non-selective/general medium and PALCAM and XLD were used as selective media to enumerate the individual cell density of *L. monocytogenes* and *S. Typhimurium* present within this dual-species biofilm. As mentioned before, on these selective media, only healthy cells are able to form colonies, so only these cells were taken into account while determining the cell density of the individual populations. Therefore, a (slight) underestimation of the total number of *L. monocytogenes* and *S. Typhimurium* cells present within the dual-species biofilm could occur as a consequence of the induction of sub-lethal injury [13,24].

Before counting the colonies, agar plates were incubated for (at least) 24 h at 30 °C (BHIA and PALCAM) or 37 °C (TSA and XLD).

### 2.6. Modelling, Parameter Estimation, and Statistical Analysis

The experimental data obtained following CAP treatment of the different model biofilms was fitted using the model of Geeraerd et al. [25], describing a microbial inactivation curve consisting of a log-linear inactivation phase and a tail (Equation (1)).

(1) $$N\left(t\right)=(N_{0}-N_{res})\cdot e^{-k_{max}\cdot t}+N_{res}$$

Here, *N*(*t*) (CFU/cm^2^) is the cell density at time *t* [min], *N*_0_ (CFU/cm^2^) is the initial cell density (*t* = 0 min), *N_res_* (CFU/cm^2^) is a more resistant subpopulation, and *k_max_* (1/min) is the maximum specific inactivation rate. Based on the difference between log_10_ *N_0_* and log_10_ *N_res_*, the final log-reduction values (following 30 min of CAP treatment) were calculated.

The parameters of the Geeraerd et al. [25] model were estimated via the minimization of the sum of squared errors (SSE), using the lsqnonlin routine of the Optimization Toolbox of Matlab version R2016a (The Mathworks, Inc., Natick, MA, USA). Standard errors of the parameter estimations were at the same time determined based on the Jacobian matrix. Finally, the Root Mean Squared Error (RMSE) value served as an absolute measure of the goodness of the model to fit the actual obtained data.

Theoretical concentrations obtained from the model of Geeraerd et al. [25] were used for both the non-selective/general and selective counts to calculate the percentage of sub-lethally injured cells (% SI) present within the single-species (*L. monocytogenes* and *S. Typhimurium*) biofilms. The equation of Busch and Donnelly [26] (Equation (2)) was used to determine the percentage of injured cells at each treatment time. As a result, the percentage of sub-lethal injury was plotted as function of the treatment time (0–30 min).

(2) $$\%\mathrm{SI}=\frac{\mathrm{CFU}\;\mathrm{non}-\mathrm{selective}\;\mathrm{medium}-\mathrm{CFU}\;\mathrm{selective}\;\mathrm{medium}}{\mathrm{CFU}\;\mathrm{non}-\mathrm{selective}\;\mathrm{medium}}\cdot 100$$

Analysis of variance (ANOVA) tests were performed to determine whether there were any significant differences between the estimated model parameters obtained following CAP treatment of each of the model biofilms, using the air-based system. A confidence level of 95.0% (α = 0.05) was applied and Fisher´s Least Significant Difference (LSD) test was used to distinguish which means were significantly different from others. In addition, different ANOVA tests were carried out to compare the presented model parameters (obtained while using the air-based set-up) with those previously observed while using a helium-operated system [12,13,14].

All statistical analyses were performed using the Statgraphics 18 software (Statistical Graphics, Washington, DC, USA). Significant differences between sample values/estimated model parameters were indicated with different (uppercase) letters or different numbers (e.g., ‘a’, ‘b’, ‘A’, ‘B’, ‘1’, ‘2’, …), with ‘a’, ‘A’ or ‘1’ indicating the lowest value.

## 3. Results and Discussion

### 3.1. Kinetics Obtained Following Biofilm Inactivation with an Air-Based CAP System

**Figure 1:**
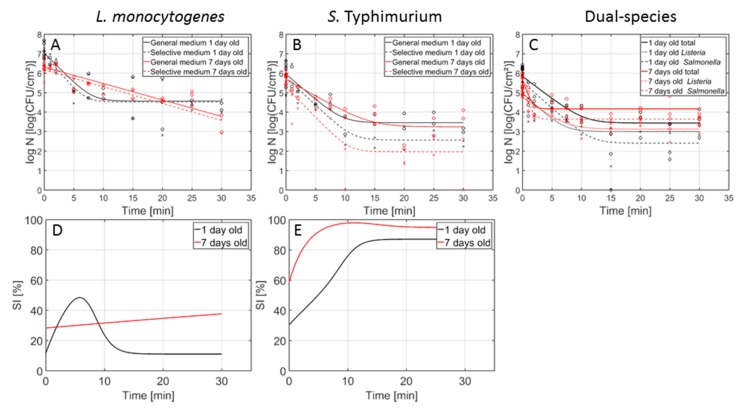
Cell density (log_10_(CFU/cm^2^)) of the 1- (black) and 7- (red) day-old *L. monocytogenes*, *S. Typhimurium*, and dual-species model biofilms as function of the CAP treatment time using an air-based cold atmospheric plasma (CAP) system (*n* = 3). Both the experimental data (symbols) and the global fit (line) of the Geeraerd et al. [25] model are represented. For the single-species (**A**) *L. monocytogenes* and (**B**) *S. Typhimurium* biofilms: total viable population on general medium (o, solid line) and uninjured viable population on selective medium (x, dashed line). For the (**C**) dual-species biofilm: total population on general medium (o, solid line), *L. monocytogenes* population on PALCAM medium (x, dash-dot line), and *S. Typhimurium* population on XLD medium (◊, dashed line). The percentage of sub-lethally injured cells (SI) as function of the CAP treatment time have been included as well for the (**D**) *L. monocytogenes* and (**E**) *S. Typhimurium* single-species model biofilms.

Figure 1 presents the inactivation curves obtained following CAP treatment of the 1 and 7-day-old (A) *L. monocytogenes*, (B) *S. Typhimurium*, and (C) dual-species model biofilms using the air-based (SBD) system. In this figure, the percentage of sub-lethal injury as function of the CAP treatment time has been included as well for the (D) *L. monocytogenes* and (E) *S. Typhimurium* single-species model biofilms. In Table 1, the corresponding model parameters of the Geeraerd et al. [25] model can be observed for each of the model biofilms following CAP treatment with the air-based (SBD) system. To determine if there were significant differences between the model parameters obtained for the different (i) biofilm ages (i.e., 1 day vs. 7 days), (ii) biofilm types (i.e., *L. monocytogenes* vs. *S. Typhimurium* vs. dual-species), or (iii) population types (i.e., *L. monocytogenes* vs. *S. Typhimurium* within the dual-species biofilm), different ANOVA tests were performed. Significant differences have been indicated with different small letters (e.g., a, b, or c), with ‘a’ bearing the lowest value.

#### 3.1.1. General Observations

In general, the inactivation curves had a similar shape, i.e., a log-linear inactivation phase was followed by a tail with a residual cell density, indicating that a prolonged CAP treatment (at the specifically applied CAP conditions) would not result in higher log-reductions. Only one exception was observed, i.e., for the 7-day-old *L. monocytogenes* biofilms, the log-linear inactivation phase was not followed by a tail. The data obtained for this specific biofilm type has been fitted using the log-linear inactivation model of Geeraerd et al. [25], which can be obtained by replacing *N_res_* in Equation (1) by 0. As a result, there were no log_10_ *N_res_* values included in Table 1 for the 7-day-old *L. monocytogenes* biofilm. Nevertheless, the remaining cell densities following 30 min of CAP treatment were used to calculate the obtained log-reductions. These remaining cell densities (based on the model fit) were 3.79 and 3.58 log_10_(CFU/cm^2^) for the non-selective/general and the selective medium, respectively. The continuous log-linear inactivation phase obtained for the 7-day-old *L. monocytogenes biofilms* indicates that higher log-reductions could still be obtained if the CAP treatment time would be further increased. Additionally, in comparison to all other CAP treated model biofilms, this could hint on a higher ability of the (reactive) plasma species to penetrate further into the three-dimensional matrix of this specific model biofilm, enabling them to interact as well with the cells located in the lower layers of the model biofilm. Nevertheless, further research (e.g., by means of confocal laser scanning microscopy (CLSM) combined with live/dead staining) would be required to confirm this hypothesis.

#### 3.1.2. Influence of the Biofilm Age

Based on Figure 1 and Table 1, it can be concluded that the biofilm age had an influence on the model parameters obtained for two biofilm types, i.e., the *L. monocytogenes* single-species biofilm and the dual-species biofilm. For the *S. Typhimurium* single-species biofilm, on the other hand, the inactivation kinetics were not influenced by the biofilm age.

For the *L. monocytogenes* single-species biofilm, higher initial cell densities (log_10_ *N_0_*) were observed for the 1 day-old biofilm. This has been observed before in the research of Govaert et al. [12] where this decrease in initial cell density at an increased biofilm age was attributed to nutrient starvation and/or waste accumulation [27]. Apart from the higher initial cell densities, higher *k_max_* values and residual cell densities (log_10_ *N_res_*) were obtained as well for the 1 day-old *L. monocytogenes* biofilms. However, for the latter model parameter, this was not significantly proven by an ANOVA test since there were no standard errors available for the remaining cell density of the 7 day-old *L. monocytogenes* biofilm following 30 min of CAP treatment (see Section 3.1.1). The higher inactivation rate and residual/remaining cell density for the 1 day-old biofilms indicate that the reactive plasma species can more easily (and quickly) penetrate into this 1 day-old model biofilm and that the biofilm-associated cells tended to become less resistant towards CAP treatment if the biofilm age increased. Nevertheless, despite a difference in residual/remaining cell density values, similar log-reduction values were obtained for both *L. monocytogenes* single-species biofilm ages. Consequently, it can be concluded that the efficacy of the air-based CAP system for *L. monocytogenes* biofilm inactivation is independent of the biofilm age, although higher log-reduction values could probably still be obtained for the 7 day-old model biofilm if a longer treatment time would be applied.

For the dual-species biofilm, similar initial cell densities were obtained for both biofilm ages. This is in contradiction to the results obtained for the single-species biofilms, indicating that during the biofilm development phase a certain form of metabolic cooperation was established between the two species present within the dual-species biofilm, i.e., one species could have provided nutrients for the other species during the 24 h period in which the two species had been living together prior to the enumeration of the initial cell density (log_10_ *N_0_*) [14,28,29]. Nevertheless, as mentioned in the study of Govaert et al. [14], other mechanisms could have been involved as well, e.g., the composition of the EPS matrix could change as function of the biofilm age, resulting in a better retention of water and/or nutrients and less starvation of the cells. For the inactivation rates, the residual cell densities, and the log-reduction values obtained for the 1 and 7 day-old dual-species biofilms, significant differences were dependent on the population type. The biofilm age had no influence on these model parameters obtained for the *L. monocytogenes* population of the dual-species biofilms. Nevertheless, for the *S. Typhimurium* biofilm-associated cells (and the total population), higher *k_max_* and log_10_ *N_res_* values were obtained for the 7 day-old biofilms. As a consequence, significantly higher log-reduction values were obtained for the *S. Typhimurium* (and the total) population present within the 1 day old dual-species biofilm. This increased resistance of the *S. Typhimurium* cells at an increased biofilm age indicates again that cooperative interactions might have been established between the *L. monocytogenes* and *S. Typhimurium* cells, providing fitness advantages for the latter species [29,30]. Nevertheless, a more profound investigation of the intra/inter-species interactions would be required to thoroughly explain the observed results.

#### 3.1.3. Influence Biofilm Type

Within this section, the model parameters obtained for the (1- and 7-day-old) dual-species biofilms using the selective media (i.e., PALCAM and XLD for the *L. monocytogenes* and *S. Typhimurium* population, respectively) were compared to the corresponding model parameters obtained for the (1- and 7-day-old) single-species biofilms while using the selective media. Based on Figure 1 and Table 1, it can be concluded that the influence of the biofilm type on the CAP inactivation kinetics was dependent on the biofilm age.

For the 1-day-old biofilms, the biofilm type generally did not have an influence on the obtained model parameters. Only for the initial and the residual cell density, the obtained model parameters were higher for the 1-day-old *L. monocytogenes* single-species model biofilm than for the *L. monocytogenes* population present with the dual-species model biofilm. The higher initial *L. monocytogenes* cell density observed for the single-species biofilm has been observed before in the research of Govaert et al. [14]. Within this study, using a helium-based (DBD) CAP set-up for dual-species biofilm inactivation, it was hypothesized that the nutrient concentration of the dual-species growth medium was not sufficient for the *L. monocytogenes* cells to reach similar densities as for the single-species biofilm, where a more nutrient-rich growth medium was applied. The higher residual cell density obtained for the *L. monocytogenes* cells present within the single-species biofilm, on the other hand, indicates that the *L. monocytogenes* cells became less resistant towards the applied CAP treatment if they were part of the dual-species biofilm. Based on this, it can be concluded that also competitive interactions were established between the cells present within the dual-species biofilm. According to the research of Elias and Banin [29], competitive interactions such as the production of bacteriocins can result in a decreased resistance towards antimicrobial agents and other inactivation technologies. Nevertheless, despite these significant differences in initial and residual cell density values, there were no significant differences between the obtained log-reduction values. Consequently, it can be concluded that the efficacy of the CAP treatment was not altered when the *L. monocytogenes* and *S. Typhimurium* cells became part of the (1-day-old) dual-species biofilm.

For the 7-day-old biofilms, the biofilm type highly influenced the obtained model parameters. For the initial cell density, again higher values were obtained for the *L. monocytogenes* single-species biofilm than for the corresponding population present within the dual-species biofilm. As for the 1-day-old biofilms, this can be the result of the limited nutrient concentration of the dual-species growth medium. Regarding the inactivation rate *k_max_*, significantly higher values were obtained for the *L. monocytogenes* and *S. Typhimurium* population present within the dual-species biofilm, hinting that the reactive plasma species can more easily (i.e., faster) penetrate into the dual-species biofilm matrix. This observed phenomenon can be explained based on an additional experiment (Figure S1) quantifying the total biofilm mass (cells + matrix) of the untreated model biofilms. As the lowest biofilm mass was observed for the 7-day-old dual-species model biofilm, this indicates that the 3-dimensional matrix of this specific model biofilm was less dense, resulting in an increased penetration rate of the (reactive) plasma species. CLSM experiments in combination with fluorescent dyes to visualize the biofilm matrix can aid to confirm this claimed hypothesis. For the residual/remaining cell density, higher values were obtained for the 7-day-old *L. monocytogenes* single-species biofilm than for the *L. monocytogenes* population present within the corresponding dual-species biofilm; although this could again not be significantly proven (see Section 3.1.1). As for the 1-day-old biofilm, the higher remaining *L. monocytogenes* cell density observed for the single-species biofilm can be explained based on the existence of competitive interactions within the dual-species biofilm. For the *S. Typhimurium* cells, the opposite trend was observed, i.e., the *S. Typhimurium* biofilm-associated cells tended to become more resistant towards CAP treatment if they were part of the dual-species biofilm. According to the study of Burmølle et al. [31], this means that the *S. Typhimurium* cells showed selfish behaviour, i.e., the (competitive) inter-species interactions resulted in a positive effect on the actor (*S. Typhimurium*), while it affected the receiver (*L. monocytogenes*) in a negative way. Finally, higher log-reduction values were obtained for the 7-day-old *L. monocytogenes* and *S. Typhimurium* single-species biofilms than for the corresponding populations present within the dual-species biofilm. For *L. monocytogenes*, this is in contradiction to the higher remaining *L. monocytogenes* cell density observed for the 1-day-old biofilms. Probably, this (contradictory) observation is caused by the difference in initial cell density between the two biofilm types and/or due to the non-statistically proven difference in residual/remaining cell density.

#### 3.1.4. Influence Population Type within Dual-Species Biofilm

Within the dual-species biofilm, the inactivation kinetics (Figure 1C and Table 1) were influenced by the population type (i.e., *L. monocytogenes* vs. *S. Typhimurium*). For the 1-day-old dual-species biofilm, the initial *S. Typhimurium* cell density was higher than for *L. monocytogenes*. In the research of Govaert et al. [14], this was explained based on the applied growth conditions during the dual-species biofilm development. As the *S. Typhimurium* and *L.* monocytogenes cells had different nutrient requirements (i.e., low vs. high concentration, respectively) to develop single-species biofilms [23], the nutrient-poor growth medium used for the dual-species biofilms was deemed to be more favourable for *S. Typhimurium* growth. The inactivation rates obtained for the 1-day-old dual-species biofilm were similar for both population types, but a significantly higher residual *L. monocytogenes* cell density was obtained. Consequently, the obtained log-reduction value was also lower for *L. monocytogenes* than for *S. Typhimurium* present within the 1-day-old dual-species model biofilm. Despite the selfish behaviour of the *S. Typhimurium* cells present within the dual-species biofilms (see Section 3.1.3), CAP treatment appeared to be more effective for inactivation of the *S. Typhimurium* cells present within the 1-day-old dual-species biofilm than for the *L. monocytogenes* cells. Nevertheless, this could also be related to the significantly lower initial *L. monocytogenes* cell density.

For the 7-day-old dual-species biofilms, initial *S. Typhimurium* cell densities were again higher than for *L. monocytogenes*. As mentioned before, this can be explained based on the environmental conditions encountered during the biofilm development. The inactivation rate and the residual cell density were as well higher for the *S. Typhimurium* population. Nevertheless, the obtained log-reduction values remained similar for both population types, indicating that the efficacy of the CAP treatment is not altered by the population type.

#### 3.1.5. Induction of Sub-Lethal Injury

Regarding the effect of the biofilm age on the induction of sub-lethal injury using the air-based SBD system (Figure 1D,E), it can be observed that the initial percentage of sub-lethal injury increased with an increased biofilm age. This has (partially) been observed before in previous research investigating the influence of the biofilm age on the CAP inactivation kinetics using a helium-based (DBD) set-up [12]. In previously mentioned study, an increased biofilm age resulted in an increased initial percentage of sub-lethal injury for *S. Typhimurium*, although this increase was rather limited (approximately 15%). The slightly higher increase in sub-lethal injury observed within the presented research could be a consequence of experimental/biological variability and/or the model fit. For *L. monocytogenes,* on the other hand, previous research proved that the biofilm age had no influence on this initial percentage of sub-lethal injury [12], while a relatively small increase of approximately 15% was observed within the presented research. Again, this could be the result of experimental/biological variability and/or the model fit. For the percentage of sub-lethal injury as function of the CAP treatment time, it can be observed that the *L. monocytogenes* biofilm age altered the shape of the curve. For the 1-day-old biofilm, an initial increase was followed by a peak and a subsequent decrease until a residual (low) percentage was obtained. In addition, the peak in sub-lethal injury coincided with the log-linear inactivation phase, indicating that a period of injury accumulation was followed by cell death [24]. For the 7-day-old *L. monocytogenes* biofilms, on the other hand, the percentage of sub-lethal injury kept on increasing as function of time, which can be related to the continuous log-linear inactivation phase observed in Figure 1A. For the *S. Typhimurium* biofilms, the curves had a similar shape for both biofilm ages, i.e., an initial increase was followed by a peak and a residual percentage of sub-lethal injury. It should be stressed, however, that this percentage was very high for both biofilm ages. Consequently, these sub-lethally injured cells are not taken into account when only selective media are used during the microbial analysis of food contact surfaces, posing a significant health risk due to an underestimation of the level of contamination.

With respect to the influence of the biofilm type on the percentage of sub-lethal injury obtained following the air-based CAP treatment, it can be concluded that the initial percentages of sub-lethal injury were higher for the *L. monocytogenes* single-species biofilms than for the corresponding *S. Typhimurium* biofilms. This has been observed before in the research of Govaert et al. [13], where this was deemed to be a consequence of the generally higher resistance of the former species towards various environmental stresses such as nutrient limitations and dehydration. For the sub-lethal injury percentage as function of the CAP treatment time, it can be observed that the residual percentage of sub-lethal injury is much higher for *S. Typhimurium* than for *L.* monocytogenes. As mentioned before, this could result in an underestimation of the *S. Typhimurium* cell density and a subsequent health risk.

### 3.2. Comparison between the Air-Based and Helium-Operated CAP System

Within this section, the results obtained following CAP treatment of the different model biofilms using the air-based (SBD) CAP system were compared to the corresponding results obtained using a helium-operated set-up. These previously obtained datasets using the helium-operated CAP system were part of a thorough investigation examining the influence of different plasma and biofilm characteristics on the CAP inactivation efficacy and kinetics. For the helium-operated treatment, two different electrode configurations were applied (see Section 2.4), i.e., an SBD and a DBD electrode. For the former configuration (SBD-helium), inactivation results were only available for the 1-day-old *L. monocytogenes* and *S. Typhimurium* single-species biofilms as these results were part of an elaborated study examining the effect of different plasma characteristics on the efficacy of CAP for inactivation of 1-day-old single-species biofilms [13]. Comparing these previously acquired results (SBD-helium) with those obtained using the air-based SBD set-up gives an indication on the importance of the applied gas composition on the biofilm inactivation efficacy of CAP. For the latter configuration (DBD-helium), results were available for all biofilm types and both biofilm ages since the combination of a DBD electrode, helium as working gas, and an input voltage of 21.88 V proved to be optimal for inactivation of the 1-day-old single-species biofilms [13], which in turn resulted in a further investigation of the efficacy of this optimal set of conditions for inactivation of older [12] and dual-species [14] model biofilms. When these previously obtained results (DBD-helium) are compared to those acquired within the presented study (SBD-air), one can comment on the importance of the electrode configuration (while also taking into account the effect of the working gas composition).

**Figure 2:**
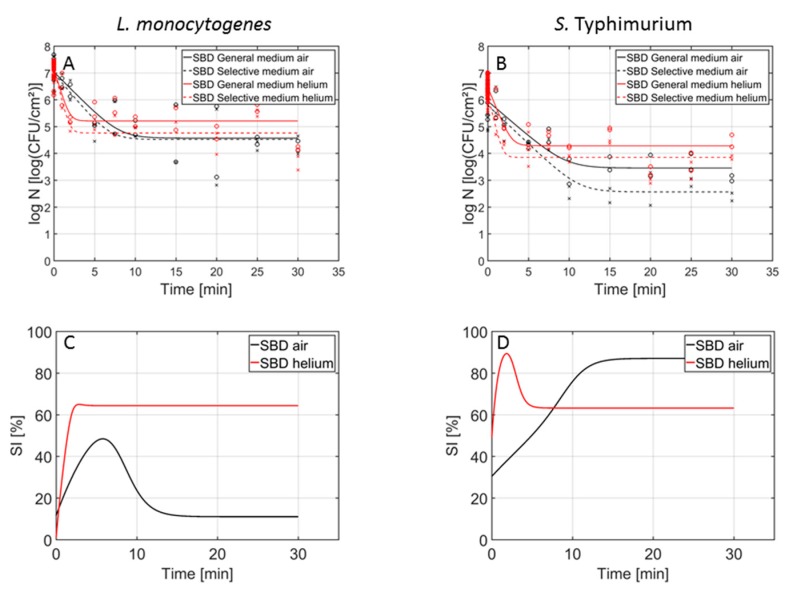
Cell density (log_10_ (CFU/cm^2^)) of the 1-day-old (**A**) *L. monocytogenes* and (**B**) *S. Typhimurium* single-species model biofilms as function of the CAP treatment time using an air-based (black) or helium-operated (red) (SBD) CAP system (*n* = 3). Both the experimental data (symbols) and the global fit (line) of the Geeraerd et al. [25] model are represented: total viable population on general medium (o, solid line) and uninjured viable population on selective medium (x, dashed line). The percentage of sub-lethally injured cells (SI) as function of the CAP treatment time have been included as well for the (**C**) *L. monocytogenes* and (**D**) *S. Typhimurium* single-species model biofilms using an air-based (black) or helium-operated (red) CAP system.

**Figure 3:**
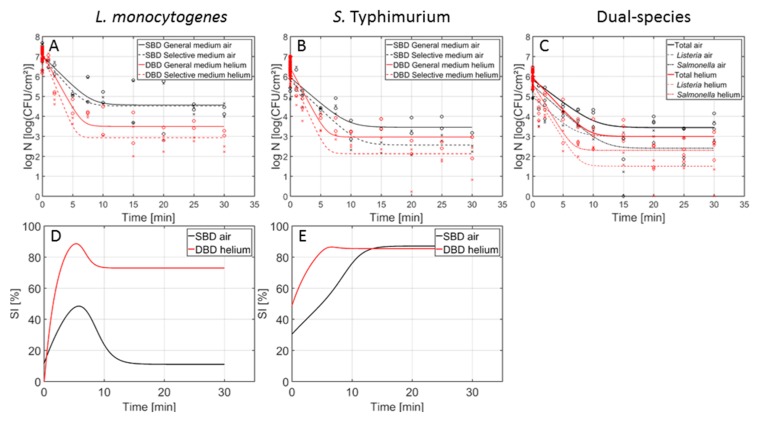
Cell density (log_10_ (CFU/cm^2^)) of the 1-day-old *L. monocytogenes*, *S. Typhimurium*, and dual-species model biofilms as function of the CAP treatment time using an air-based (black) or helium-operated (red) CAP system (n = 3). Both the experimental data (symbols) and the global fit (line) of the Geeraerd et al. [25] model are represented. For the single-species (**A**) *L. monocytogenes* and (**B**) *S. Typhimurium* biofilms: total viable population on general medium (o, solid line) and uninjured viable population on selective medium (x, dashed line). For the (**C**) dual-species biofilm: total population on general medium (o, solid line), *L. monocytogenes* population on PALCAM medium (x, dash=dot line), and *S. Typhimurium* population on XLD medium (◊, dashed line). The percentage of sub-lethally injured cells (SI) as function of the CAP treatment time have been included as well for the (**D**) *L. monocytogenes* and (**E**) *S. Typhimurium* single-species model biofilms using an air-based (black) or helium-operated (red) CAP system.

**Figure 4:**
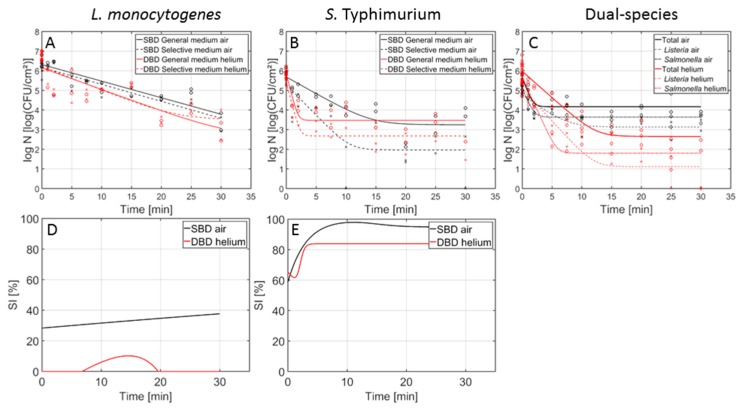
Cell density (log_10_ (CFU/cm^2^)) of the 7-day-old *L. monocytogenes*, *S. Typhimurium*, and dual-species model biofilms as function of the CAP treatment time using an air-based (black) or helium-operated (red) CAP system (n = 3). Both the experimental data (symbols) and the global fit (line) of the Geeraerd et al. [25] model are represented. For the single-species (**A**) *L. monocytogenes* and (**B**) *S. Typhimurium* biofilms: total viable population on general medium (o, solid line) and uninjured viable population on selective medium (x, dashed line). For the (**C**) dual-species biofilm: total population on general medium (o, solid line), *L. monocytogenes* population on PALCAM medium (x, dash-dot line), and *S. Typhimurium* population on XLD medium (◊, dashed line). The percentage of sub-lethally injured cells (SI) as function of the CAP treatment time have been included as well for the (**D**) *L. monocytogenes* and (**E**) *S. Typhimurium* single-species model biofilms using an air-based (black) or helium-operated (red) CAP system.

In Figure 2, the inactivation curves obtained following CAP treatment of the 1-day-old (A) *L. monocytogenes* and (B) *S. Typhimurium* single-species model biofilms using the air- and helium-operated SBD set-up can be observed. In addition, in Figure 2C,D, the percentage of sub-lethal injury as function of the CAP treatment time (using both CAP systems) has been included for *L. monocytogenes* and *S. Typhimurium*, respectively. In Figure 3 (1 day old biofilms) and Figure 4 (7 days old biofilms), on the other hand, the inactivation curves obtained following CAP treatment of the (A) *L. monocytogenes*, (B) *S. Typhimurium*, and (C) dual-species model biofilms using the air-based SBD and the helium-operated DBD set-up can be observed. In Figure 3D,E and Figure 4D,E, the percentages of sub-lethal injury as function of the CAP treatment time have again been included for the (1- and 7-day-old) *L. monocytogenes* and *S. Typhimurium* biofilms, respectively. As mentioned before, Table 1 presents the model parameters obtained following treatment of the different model biofilms with the air-based (SBD) CAP system. Table 2 and Table 3, on the other hand, include the corresponding parameters obtained following biofilm treatment with the helium-operated SBD and DBD set-ups, respectively. For each of the model parameters, a separate ANOVA test has been performed to determine if there were significant differences between the values obtained with the air-based (SBD) set-up (Table 1) and the helium-operated SBD (Table 2) or DBD (Table 3) system. Significant differences have been indicated with a different number (SBD-helium vs. SBD-air) or a different upper-case letter (DBD-helium vs. SBD-air), with ‘1’ and ‘A’ bearing the lowest values.

#### 3.2.1. General Observations

Based on Figure 2, Figure 3 and Figure 4, it can be concluded that changing the working gas composition and/or the electrode configuration did not result in an altered shape of the inactivation curves. If a log-linear inactivation phase was followed by a tail phase for a certain model biofilm, this was the case for both electrode configurations and both working gas compositions. Similarly, if the inactivation curve only contained a log-linear phase (i.e., for the 7-day-old *L. monocytogenes* single-species model biofilm), this was observed for both available datasets (i.e., DBD-helium and SBD-air).

#### 3.2.2. Differences in Inactivation Kinetics between SBD-air and SBD-helium

Based on Table 1, Table 2 and Figure 2, several observations can be done regarding the effect of the working gas composition on the CAP inactivation kinetics for the 1-day-old *L. monocytogenes* and *S. Typhimurium* single-species model biofilms.

First of all, the initial cell density involves the cell density of the untreated biofilms, which were always developed at the same conditions. As a consequence, obtained differences in initial *L. monocytogenes* and *S. Typhimurium* cell densities should be allocated to experimental/biological variability and/or the model fit.

With respect to the inactivation rate, significantly higher values were always obtained using the helium-operated SBD set-up. Based on this, it can be concluded that the (reactive) plasma species generated using this helium-operated CAP system were able to more easily (i.e., faster) penetrate into the biofilm matrix and/or resulted in a faster inactivation of the biofilm-associated cells. However, the inactivation rate is not the only important parameter, i.e., the duration of the log-linear inactivation phase is also very important since no further inactivation is obtained beyond this point. Based on Figure 2, it can be concluded that the use of helium resulted in a much shorter log-linear inactivation phase (<5 min) in comparison to the air-based system (10–15 min). Thus, despite the higher inactivation rate reported using helium as working gas, the use of air could be more favourable since these species are most likely able to penetrate further into the deeper layers of the biofilms.

Regarding the residual cell densities, (significantly) lower residual cell densities were obtained using the air-based system. Consequently, the biofilm-associated cells tended to be more resistant towards the (reactive) plasma species generated using the helium-based set-up. Nevertheless, when the obtained log-reductions were compared, both SBD systems resulted in a similar reduction in the initial cell density, although the values obtained using the air-based set-up were generally higher than those obtained using the helium-operated system. However, this difference was only significant for the *S. Typhimurium* model biofilm using the selective medium.

According to Bourke et al. [20] and Ragni et al. [32], the gas composition used for plasma generation is one of the most important influencing plasma characteristics, as the applied feed gas (and its relative humidity) mainly determines the range and the type of generated (reactive) species. Comparing the two SBD systems (i.e., SBD-air vs. SBD-helium) therefore reveals the impact of this feed gas composition on the antimicrobial efficacy of the CAP treatment. Using the SBD configuration, no direct contact is made between the sample and the plasma. As a result, the reactive species first have to travel through a region of neutral gas before they can potentially reach the sample. Species transport in an SBD system is governed by diffusive and convective processes, with the latter being the result of electro-hydrodynamic forces produced by the movement of charged particles in the plasma [33,34]. It is well known that highly reactive species (e.g., ions, electronically excited states, and reactive neutrals such as atomic oxygen) are unable to propagate beyond the visible plasma region. At the plasma-gas interface, they react to form less reactive and more stable RONS (e.g., O + O_2_ → O_3_), which are primarily responsible for the inactivation effect observed.

Therefore, when comparing the impact of the gas composition, the quenching of RONS by the background gas must be considered. In a helium-rich atmosphere the production of RONS in the plasma region is limited by the availability of precursor molecules (e.g., O_2_, N_2_ and H_2_O originating from contaminants of the feed gas or penetration of (humid) air through feed gas tubes or other plastic material [35]). However, the quenching rate of the generated RONS is comparatively low, hence some highly reactive species such as OH could potentially reach the sample in a concentration sufficient to play a role in the microbial inactivation. Conversely, air-based SBD’s are able to generate large densities of RONS within the plasma region, yet these species react quickly at the plasma–gas interface resulting in only the less reactive (intermediate) species that are able to reach the sample. Past studies on air based SBD’s have revealed that only seven species (O_3_, NO, N_2_O, NO_2_, N_2_O_5_, HNO_2_, and HNO_3_) out of the potential 65 species generated in the plasma region are able to propagate >10 mm from the visible discharge at levels exceeding 10 ppm.

Although the specifically generated plasma species have not been measured within the presented study, the generally higher log-reduction values obtained using the air-based system indicate that mainly the high nitrogen content of the air is beneficial to obtain a high biofilm inactivation efficacy since the study of Govaert et al. [13] indicated that the addition of 1% (*v/v*) oxygen to a helium feed gas did not result in an increased inactivation efficacy. As mentioned before, the study of Duan et al. [22] proved that RNS are able to penetrate further into biological tissue, enabling them to inactivate cells located in the lower layers of the biofilm. In addition, many RNS such as nitrite, nitrate, and peroxynitrite proved to have a (strong) bactericidal effect (e.g., [36,37,38,39]). Nevertheless, more research is required to confirm this hypothesis.

#### 3.2.3. Differences in Inactivation Kinetics between SBD-air and DBD-helium

Within this section, the inactivation kinetics obtained using the air-based SBD system were compared to those acquired using the helium-based DBD system. Since the effect of the operating gas on the efficacy of the CAP treatment proved to be rather limited (see Section 3.2.2), possible significant differences in inactivation efficacy observed between the SBD-air and the DBD-helium set-up can be mainly attributed to a difference in electrode configuration/exposure mode (DBD = direct treatment, SBD = indirect treatment), rather than to a difference in operating gas. Nevertheless, one should be careful interpreting these results as also the frequency, the plasma power, and the output voltage were not exactly the same for both CAP systems.

Although the general shape of the curves was retained when the electrode configuration was altered (Figure 3 and Figure 4), some of the model parameters (Table 1 and Table 3) were influenced by this important plasma characteristic.

For the initial cell density of the biofilms, in general no significant differences were observed. As mentioned in previous section, minor differences in initial cell density values can be attributed to experimental/biological variability and/or the model fit.

Regarding the inactivation rate, values obtained with the helium-based DBD system were generally higher than those obtained with the air-based SBD system. Nevertheless, this was not significantly demonstrated. Only one exception was observed, i.e., for the 7-day-old dual-species biofilm, higher values were obtained with the air-based system. Comparing the duration of the log-linear inactivation phases, both electrode configurations resulted in a similar duration (i.e., approximately 10 min) for the 1-day-old biofilms, while for the 7-day-old biofilms, the effect of the electrode configuration on this value was dependent on the biofilm type. Based on previously mentioned observations, it can be concluded that the DBD system generally resulted in the creation of (reactive) plasma species which were able to penetrate faster into the biofilm matrix. This can be the result of the difference in exposure mode between both electrode configurations, i.e., direct vs. remote.

For the residual cell densities, in general higher values were obtained following treatment with the air-based SBD system, although these differences were not always significantly proven. This observation indicates that the biofilm-associated cells tended to be (slightly) more resistant towards the plasma species generated using the air-based SBD system. As a consequence, the highest log-reduction values were in general obtained using the helium-based DBD system.

While the three investigated systems (SBD-air, SBD-helium and DBD-helium) are all based on the dielectric barrier discharge principal, the underpinning physicochemical pathways of how reactive species generated within the plasma reach and interact with the biofilm differ significantly. From a theoretical perspective, the DBD system should offer the highest performance in terms of its microbial inactivation efficacy. In this direct-contact scenario, the biofilm is placed directly between the plasma generating electrodes and is thus exposed to large densities of highly energetic RONS, including charged particles and short-lived radicals such as atomic oxygen. In contrast, all SBD based systems rely on the transport of RONS from the plasma to the sample, providing an opportunity for secondary reactions to occur [21,40]. Figure 3 and Figure 4 confirm this, showing higher levels of inactivation after a shorter treatment time with the direct-contact DBD system compared to the indirect-contact SBD system.

#### 3.2.4. Induction of Sub-Lethal Injury

Comparing the percentages of sub-lethal injury obtained using the air- and helium-based SBD set-ups (Figure 2C,D), it can be concluded that the shape of the curves is slightly influenced by the working gas composition. Dependent on the specific biofilm type and working gas, the initial percentage in sub-lethal injury and the subsequent increase was either followed by a decrease and a residual percentage, or the peak percentage equalled the residual percentage. For the *L. monocytogenes* single-species biofilm, air resulted in the lowest residual percentage of sub-lethal injury, while for *S. Typhimurium*, helium proved to be more optimal. Consequently, following CAP treatment of biofilms (using any type of gas), it is important to take into account that the level of contamination might have been underestimated.

Regarding the influence of the electrode configuration (Figure 3D,E and Figure 4D,E), the shape of the curves was generally not influenced by the applied set-up. Only for the 7-day-old *L. monocytogenes* biofilms, the percentage of sub-lethal injury using the air-based SBD system kept on increasing, while for the helium-based DBD treatment, the small peak was followed by a decrease until a value of 0% was reached. The electrode configuration had, however, an influence on the residual percentages of sub-lethal injury. In general, (slightly) higher values were obtained using the (air-based) SBD set-up. Only for the 1-day-old *L. monocytogenes* biofilm, the (helium-based) DBD resulted in the highest underestimation of the contamination level. Nevertheless, one should be careful interpreting these results as other CAP characteristics were altered as well.

## 4. Conclusions

Based on previous paragraphs, it can be concluded that the efficacy of the air-based CAP treatment was influenced by the complexity of the biofilm and the population type, but not by the biofilm age. Overall log-reduction values (on non-selective/general media) ranged between 1.51 and 2.53 log_10_(CFU/cm^2^). Nevertheless, a high percentage of these cells were sub-lethally injured, which should be taken into account while assessing the level of contamination of surfaces following CAP treatment. Comparing the efficacy of the air-based SBD set-up with those obtained using the helium-based SBD and DBD set-ups proved that the efficacy of the CAP treatment was more influenced by the electrode configuration than by the operating gas. Therefore, air could be considered as a suitable alternative for helium as an operating gas, although the use of a DBD electrode configuration could help to further improve the efficacy of the treatment for biofilm inactivation. It should be stressed, however, that CAP treatment alone (using both investigated set-ups) was not sufficient to completely inactivate the biofilm-associated cells. To overcome this, the CAP treatment could be included into an entire cleaning process, using different (mild) inactivation methods/technologies. For the helium-operated system, this combined treatment principle already proved to be highly effective in reducing the bacterial cell level of the single-species biofilms to below the detection limit by combining CAP with a mild hydrogen peroxide treatment [41]. In future research, this hurdle principle can be further optimized and/or other combinations could be investigated.

## Figures and Tables

**Table 1:** Estimated model parameters of the Geeraerd et al. (2000) model obtained following inactivation of the 1 and 7 day(s)-old *L. monocytogenes*, *S. Typhimurium*, and dual-species model biofilms using the air-based SBD system.

	Single-Species *L. monocytogenes* 1-day-old	Single-Species *L. monocytogenes* 7-day-old	Single-Species *S. Typhimurium* 1 Day Old	Single-Species *S. Typhimurium* 7-day-old	Dual-Species 1-day-old	Dual-Species 7-day-old
^2^_3_ Log_10_ *N_0_* general medium ^1^_5_^4^ (log(CFU/cm^2^))	^b^ 7.04 ± 0.23 ^b^_A_^1^	^a^ 6.32 ± 0.11 ^b^_A_	^a^ 5.96 ± 0.17 ^a^_A_^1^	^a^ 5.69 ± 0.18 ^a^_A_	^a^_b_ 5.84 ± 0.21 ^a^_A_	^a^_c_ 5.69 ± 0.16 ^a^_A_
^2^_3_ Log_10_ *N_0_* PALCAM medium ^1^_5_^4^ (log(CFU/cm^2^))	^b^ 6.99 ± 0.25 ^b^_A_^1^	^a^ 6.18 ± 0.10 ^b^_A_	NA	NA	^a^_a_ 5.00 ± 0.23 ^a^_A_	^a^_a_ 4.79 ± 0.16 ^a^_A_
^2^_3_ Log_10_ *N_0_* XLD medium ^1^_5_^4^ (log(CFU/cm^2^))	NA	NA	^a^ 5.80 ± 0.20 ^a^_A_^1^	^a^ 5.31 ± 0.33 ^a^_A_	^a^_b_ 5.56 ± 0.30 ^a^_A_	^a^_b_ 5.11 ± 0.13 ^a^_A_
^2^_3_*k_max_* general medium ^1^_5_^4^ (1/min)	^b^ 0.705 ± 0.192 ^a^_A_^1^	^a^ 0.195 ± 0.018 ^a^_A_	^a^ 0.605 ± 0.116 ^a^_A_^1^	^a^ 0.396 ± 0.085 ^a^_A_	^a^_a_ 0.529 ± 0.131 ^a^_A_	^b^_b_ 2.002 ± 0.661 ^b^_B_
^2^_3_*k_max_* PALCAM medium ^1^_5_^4^ (1/min)	^b^ 0.826 ± 0.265 ^a^_A_^1^	^a^ 0.199 ± 0.017 ^a^_A_	NA	NA	^a^_a_ 0.650 ± 0.227 ^a^_A_	^a^_a_ 0.517 ± 0.159 ^b^_A_
^2^_3_*k_max_* XLD medium ^1^_5_^4^ (1/min)	NA	NA	^a^ 0.663 ± 0.114 ^a^_A_^1^	^a^ 0.722 ± 0.195 ^a^_A_	^a^_a_ 0.618 ± 0.160 ^a^_A_	^b^_b_ 1.702 ± 0.482 ^b^_A_
^2^_3_ Log_10_ *N_res_* general medium ^1^_5_^4^ (log(CFU/cm^2^))	4.57 ± 0.22 ^b^_B_^1^	/	^a^ 3.46 ± 0.17 ^a^_B_^1^	^a^ 3.25 ± 0.22 ^a^_A_	^a^_b_ 3.44 ± 0.22 ^a^_A_	^b^_c_ 4.17 ± 0.10 ^b^_B_
^2^_3_ Log_10_ *N_res_* PALCAM medium ^1^_5_^4^ (log(CFU/cm^2^))	4.52 ± 0.23 ^b^_B_^1^	/	NA	NA	^a^_b_ 3.01 ± 0.21 ^a^_A_	^a^_a_ 3.13 ± 0.16 _B_
^2^_3_ Log_10_ *N_res_* XLD medium ^1^_5_^4^ (log(CFU/cm^2^))	NA	NA	^a^ 2.57 ± 0.22 ^a^_B_^1^	^a^ 1.96 ± 0.35 ^a^_A_	^a^_a_ 2.41 ± 0.32 ^a^_A_	^b^_b_ 3.64 ± 0.09 ^b^_B_
^2^_3_ Log-reduction general medium ^1^_5_^4^ (log(CFU/cm^2^))	^a^ 2.47 ± 0.32 ^a^_A_^1^	≈ ^a^ 2.53 ± 0.12 ^b^_A_	^a^ 2.50 ± 0.24 ^a^_A_^1^	^a^ 2.44 ± 0.28 ^b^_A_	^b^_a_ 2.40 ± 0.31 ^a^_A_	^a^_a_ 1.51 ± 0.19 ^a^_A_
^2^_3_ Log-reduction PALCAM medium ^1^_5_^4^ (log(CFU/cm^2^))	^a^ 2.47 ± 0.34 ^a^_A_^1^	≈ ^a^ 2.60 ± 0.11 ^b^_A_	NA	NA	^a^_a_ 1.98 ± 0.30 ^a^_A_	^a^_a_ 1.66 ± 0.23 ^a^_A_
^2^_3_ Log-reduction XLD medium ^1^_5_^4^ (log(CFU/cm^2^))	NA	NA	^a^ 3.23 ± 0.30 ^a^_A_^2^	^a^ 3.34 ± 0.49 ^b^_A_	^b^_b_ 3.15 ± 0.44 ^a^_A_	^a^_a_ 1.47 ± 0.16 ^a^_A_
RMSE general medium (/)	0.653	0.396	0.490	0.532	0.615	0.389
RMSE PALCAM medium (/)	0.716	0.371	NA	NA	0.625	0.464
RMSE XLD medium (/)	NA	NA	0.602	0.988	0.878	0.331

^1^ Influence biofilm type (dual-species vs. single-species): for each biofilm age and each population type, model parameters bearing different superscripts (no small letters in common) are significantly different (*p* ≤ 0.05); ^2^ Influence biofilm age: for each population and biofilm type, model parameters bearing different superscripts (no small letters in common) are significantly different (*p* ≤ 0.05); ^3^ Influence population type within dual-species biofilm: for each biofilm age, model parameters bearing different subscripts (no small letters in common) are significantly different (*p* ≤ 0.05); ^4^ Influence CAP set-up (SBD-air versus SBD-helium): for each biofilm type and population type, model parameters bearing different superscripts (no numbers in common) are significantly different (*p* ≤ 0.05); ^5^ Influence CAP set-up (SBD-air versus DBD-helium): for each biofilm age, biofilm type, and population type, model parameters bearing different subscripts (no capital letters in common) are significantly different (*p* ≤ 0.05).

**Table 2:** Estimated model parameters of the Geeraerd et al. [25] model obtained following inactivation of the 1-day-old *L. monocytogenes* and *S. Typhimurium* single-species model biofilms using the helium-operated SBD system. These model parameters have been discussed in detail in the research of Govaert et al. [13].

	Single-Species *L. monocytogenes* 1-day-old	Single-Species *S. Typhimurium* 1-day-old
Log_10_ *N_0_* general medium ^4^ (log(CFU/cm^2^))	7.17 ± 0.07 ^1^	6.49 ± 0.07 ^2^
Log_10_ *N_0_* PALCAM medium ^4^ (log(CFU/cm^2^))	7.16 ± 0.07 ^1^	NA
Log_10_ *N_0_* XLD medium ^4^ (log(CFU/cm^2^))	NA	6.19 ± 0.07 ^2^
*k_max_* general medium ^4^ (1/min)	2.343 ± 0.577 ^2^	1.747 ± 0.351 ^2^
*k_max_* PALCAM medium ^4^ (1/min)	2.771 ± 0.491 ^2^	NA
*k_max_* XLD medium ^4^ (1/min)	NA	2.876 ± 0.584 ^2^
Log_10_ *N_res_* general medium ^4^ (log(CFU/cm^2^))	5.22 ± 0.11 ^2^	4.29 ± 0.11 ^2^
Log_10_ *N_res_* PALCAM medium ^4^ (log(CFU/cm^2^))	4.77 ± 0.11 ^1^	NA
Log_10_ *N_res_* XLD medium ^4^ (log(CFU/cm^2^))	NA	3.86 ± 0.11 ^2^
Log-reduction general medium ^4^ (log(CFU/cm^2^))	1.95 ± 0.14 ^1^	2.19 ± 0.13 ^1^
Log-reduction PALCAM medium ^4^ (log(CFU/cm^2^))	2.40 ± 0.13 ^1^	NA
Log-reduction XLD medium ^4^ (log(CFU/cm^2^))	NA	2.33 ± 0.13 ^1^
RMSE general medium (/)	0.432	0.396
RMSE PALCAM medium (/)	0.394	NA
RMSE XLD medium (/)	NA	0.425

^4^ Influence CAP set-up (SBD-air versus SBD-helium): for each biofilm type and population type, model parameters bearing different superscripts (no numbers in common) are significantly different (*p* ≤ 0.05).

**Table 3:** Estimated model parameters of the Geeraerd et al. [25] model obtained following inactivation of the 1- and 7-day-old *L. monocytogenes*, *S. Typhimurium*, and dual-species model biofilms using the helium-operated DBD system. These model parameters have been discussed in detail in the studies of Govaert et al. [13,14].

	Single-Species *L. monocytogenes* 1-day-old	Single-Specie *L. monocytogenes* 7-day-old	Single-Species *S. Typhimurium* 1-day-old	Single-Species *S. Typhimurium* 7-day-old	Dual-Species 1-day-old	Dual-Species 7-day-old
Log_10_ *N_0_* general medium _5_ (log(CFU/cm^2^))	7.15 ± 0.07 _A_	6.15 ± 0.22 _A_	6.43 ± 0.07 _B_	5.86 ± 0.23 _A_	5.95 ± 0.15 _A_	5.98 ± 0.13 _A_
Log_10_ *N_0_* PALCAM medium _5_ (log(CFU/cm^2^))	7.15 ± 0.07 _A_	6.22 ± 0.24 _A_	NA	NA	5.30 ± 0.21 _A_	5.09 ± 0.19 _A_
Log_10_ *N_0_* XLD medium _5_ (log(CFU/cm^2^))	NA	NA	6.14 ± 0.09 _A_	5.40 ± 0.25 _A_	5.63 ± 0.20 _A_	5.69 ± 0.24 _B_
*k_max_* general medium _5_ (1/min)	1.265 ± 0.142 _B_	0.253 ± 0.057 _A_	1.308 ± 0.154 _B_	2.730 ± 0.785 _B_	0.748 ± 0.117 _A_	0.618 ± 0.072 _A_
*k_max_* PALCAM medium _5_ (1/min)	1.735 ± 0.172 _B_	0.278 ± 0.078 _A_	NA	NA	1.118 ± 0.199 _A_	0.756 ± 0.121 _A_
*k_max_* XLD medium _5_ (1/min)	NA	NA	1.534 ± 0.198 _B_	2.598 ± 0.687 _B_	1.148 ± 0.233 _B_	1.542 ± 0.331 _A_
Log_10_ *N_res_* general medium _5_ (log(CFU/cm^2^))	3.50 ± 0.13 _A_	2.68 ± 1.71	2.97 ± 0.13 _A_	3.47 ± 0.16 _A_	3.00 ± 0.17 _A_	2.65 ± 0.16 _A_
Log_10_ *N_res_* PALCAM medium _5_ (log(CFU/cm^2^))	2.94 ± 0.13 _A_	3.50 ± 0.56	NA	NA	1.51 ± 0.23 _A_	1.12 ± 0.24 _A_
Log_10_ *N_res_* XLD medium _5_ (log(CFU/cm^2^))	NA	NA	2.13 ± 0.16 _A_	2.68 ± 0.17 _B_	2.30 ± 0.20 _A_	1.80 ± 0.23 _A_
Log-reduction general medium _5_ (log(CFU/cm^2^))	3.64 ± 0.15 _B_	3.47 ± 1.72 _A_	3.46 ± 0.14 _B_	2.38 ± 0.28 _A_	2.95 ± 0.23 _A_	3.32 ± 0.21 _B_
Log-reduction PALCAM medium _5_ (log(CFU/cm^2^))	4.22 ± 0.15 _B_	2.72 ± 0.61 _A_	NA	NA	3.79 ± 0.31 _B_	3.97 ± 0.31 _B_
Log-reduction XLD medium _5_ (log(CFU/cm^2^))	NA	NA	4.01 ± 0.18 _B_	2.72 ± 0.30 _A_	3.33 ± 0.28 _A_	3.89 ± 0.33 _B_
RMSE general medium (/)	0.435	0.733	0.420	0.571	0.500	0.439
RMSE PALCAM medium (/)	0.433	0.762	NA	NA	0.704	0.658
RMSE XLD medium (/)	NA	NA	0.547	0.626	0.646	0.784

^5^ Influence CAP set-up (SBD-air versus DBD-helium): for each biofilm age, biofilm type, and population type, model parameters bearing different subscripts (no capital letters in common) are significantly different (*p* ≤ 0.05).

## Supplementary Materials

The following are available online at https://www.mdpi.com/2304-8158/9/2/157/s1, Figure S1: Total biomass of the different model biofilms.
