# Supplementary figures and images for: Inactivation of L. monocytogenes and S. typhimurium Biofilms by Means of an Air-Based Cold Atmospheric Plasma (CAP) System
Source: Foods. 2020 Feb 6;9(2):157. doi: 10.3390/foods9020157 (PMC7074369; doi:10.3390/foods9020157)

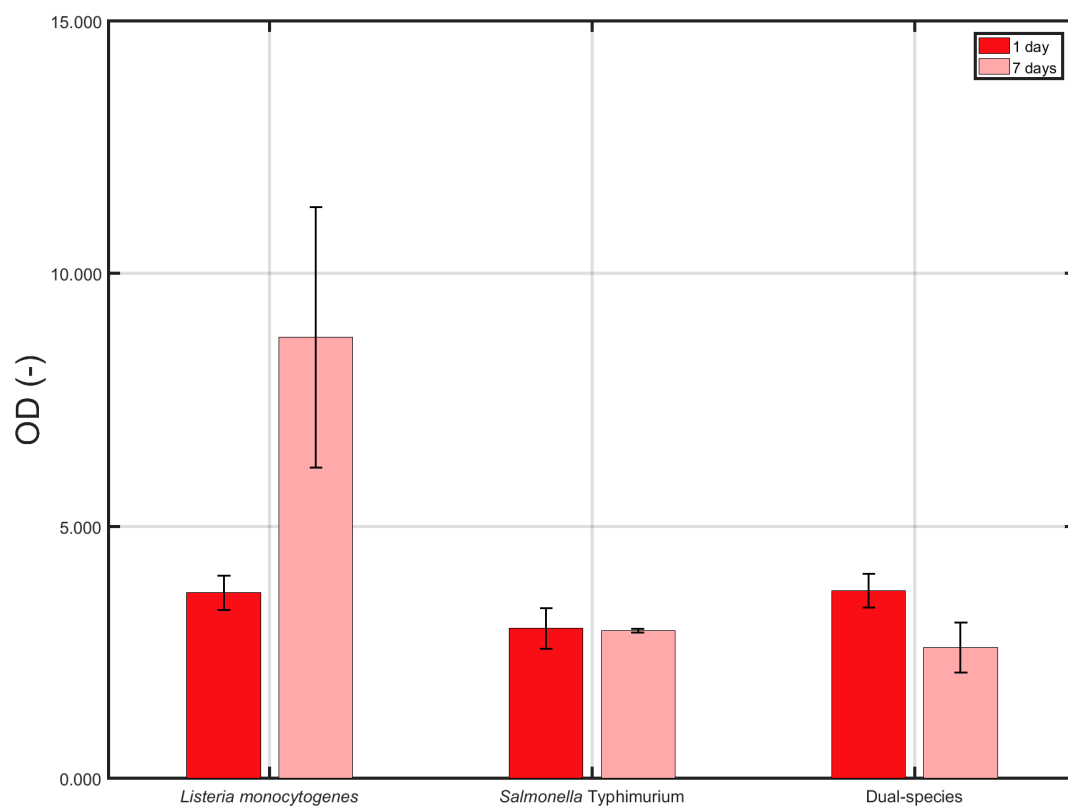

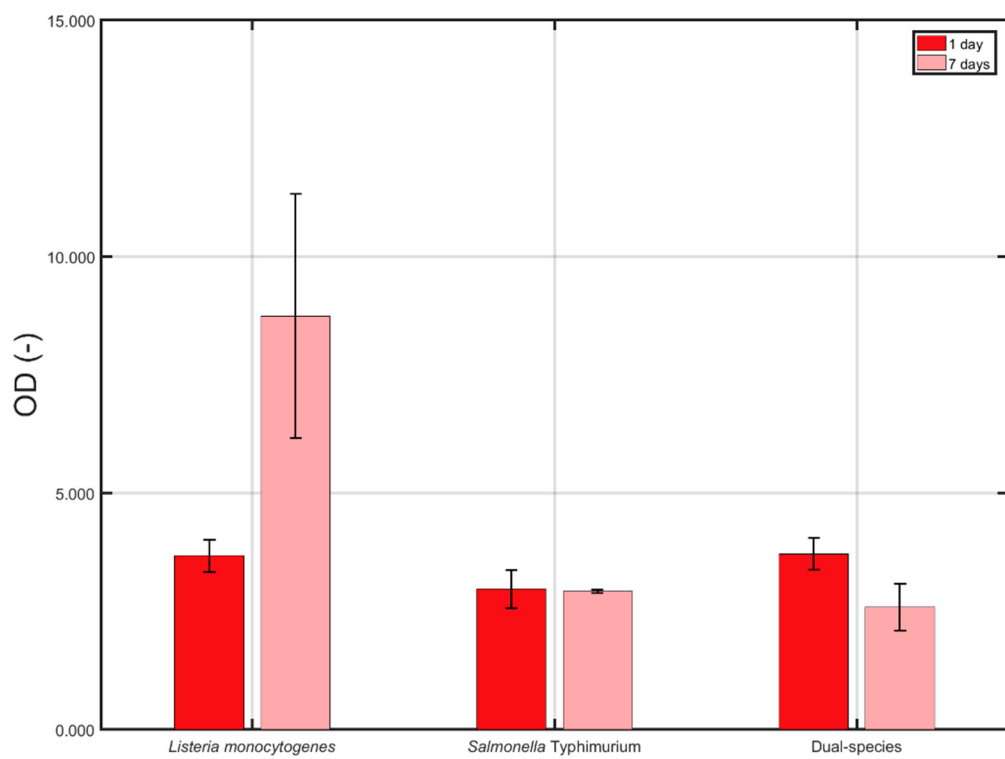

Supplement: Supplementary file 1 [file foods-09-00157-s001.pdf]
